# Sun exposure in pigs increases the vitamin D nutritional quality of pork

**DOI:** 10.1371/journal.pone.0187877

**Published:** 2017-11-14

**Authors:** D. Enette Larson-Meyer, Bennett C. Ingold, Samanta R. Fensterseifer, Kathleen J. Austin, Perry J. Wechsler, Bruce W. Hollis, Andrew J. Makowski, Brenda M. Alexander

**Affiliations:** 1 Department of Family and Consumer Sciences (Human Nutrition), University of Wyoming, Laramie, Wyoming, United States of America; 2 Department of Animal Science, University of Wyoming, Laramie, Wyoming, United States of America; 3 Department of Atmospheric Science, University of Wyoming, Laramie, Wyoming, United States of America; 4 Department of Pediatrics, Medical University of South Carolina, Charleston, South Carolina, United States of America; 5 Heartland Assays, Ames, Iowa, United States of America; INIA, SPAIN

## Abstract

There is a high prevalence of vitamin D insufficiency and deficiency worldwide likely because of both limited sun-exposure and inadequate dietary intake. Meat, including pork, is not typically considered a dietary source of vitamin D, possibly because of management practices that raise pigs in confinement. This experiment determined the vitamin D content of loin and subcutaneous adipose tissue in sun-exposed finisher pigs. Two separate groups of pigs were used. The first group (28 white Landrace-Duroc) was assigned at random to either sunlight exposure (SUN) in spring and summer or confinement per standard practice (Control). The second (24 Yorkshire-Duroc-Landrace) underwent the same exposure protocol but was exposed in summer and fall or assigned to control (Control). A subsample of five SUN and four Control pigs, matched for weight and body condition score, was selected for slaughter from each group. Pigs (n = 10 SUN, n = 8 Control) had blood drawn for analysis of 25(OH)D_3_ concentration before/after sun exposure or control, and tissue samples were taken at slaughter for analysis of tissue vitamin D3 and 25(OH)D_3_ concentration. Three random samples from a single loin chop and surrounding adipose were collected and analyzed. Serum concentrations of 25(OH)D_3_ did not differ (P≥0.376) between treatments prior to sun exposure in either group, but was increased (time*treatment interaction, P<0.001) with SUN exposure. Total vitamin D content (D3 plus 25(OH)D_3_) of loin tissue was increased (P < 0.001) with sun exposure and averaged 0.997±0.094 μg/100g and 0.348±0.027 μg/100g for sun and control pigs, respectively. While exposure to sunlight increased (P = 0.003) tissue content of 25(OH) D in subcutaneous adipose tissue, vitamin D_3_ content was similar between treatments (P = 0.56). Sunlight exposure in pigs increased the vitamin D content of loin, and may provide an additional source of dietary vitamin D.

## Introduction

Vitamin D is known for its critical role in bone health [1]. Increasing evidence also suggests vitamin D plays a role in the prevention of many chronic diseases including certain cancers [2,3,4], cardiovascular disease [5], type 2 diabetes [6], and Alzheimer’s disease [7]. Of the 30 leading causes of death in the US in 2010 19 were linked to low vitamin D status [8]. At the same time, a high prevalence of vitamin D insufficiency worldwide has been identified [9,10].

Vitamin D is unique among vitamins in that it can be obtained from diet as well as synthesized in the body from exposure to UVB radiation from sunlight. Endogenous synthesis, however, is considered the most available source of vitamin D_3_ [11]. Compared to what is typically consumed in the diet, higher amounts of vitamin D can be obtained from sun exposure. It is estimated, for example, that a single 10–15 minute [12] exposure during peak sunlight in July in a bathing suit will produce between 10,000 and 20,000 IU of vitamin D [13]. Concerns of increased risk of skin cancer and/or photo-aging, however, keep many people out of the sun. To achieve sufficiency, these individuals must obtain vitamin D through diet and/or supplementation. Food sources of vitamin D include oily fish, egg yolks, sun-dried mushrooms, and fortified milk, yogurt, margarine and several ready-to-eat cereals [14].

Meat, in general, is not considered a good source of vitamin D. The vitamin D content of pork may be particularly low because of husbandry practices, which confine pigs indoors with limited exposure to sunlight. Furthermore, the USDA nutrient database only evaluates vitamin D_3_ and D_2_ content of food sources [15]. Heaney et al. has recently argued that vitamin D content in meat products is underestimated because of the failure to consider the hydroxylated form, 25(OH)D_3_ [16], the immediate product of liver hydroxylation of vitamin D_3_ and D_2_. Later work from Heaney has suggested that solar inputs of vitamin D are less than previously thought. Instead, there is a significant input of vitamin D from undocumented food sources such as preformed 25(OH)D_3_ in animal products[17]. Moreover, 25(OH)D_3_ is estimated to be about five times as potent as vitamin D_3_ in raising serum 25(OH)D_3_ concentrations [18]. Recent tissue culture and animal studies highlight that vitamin D_3_ is readily sequestered by adipose tissue [19], while 25(OH)D_3_ is distributed throughout the body and is taken up by skeletal muscle tissue [16,20].

Although swine are generally raised in confinement, they [21], like other agricultural animals including cattle [22] and chickens [23], have the capacity to synthesize vitamin D. Sun exposure in pigs, therefore, has the potential to increase the vitamin D content in pork. The objective of this experiment was to determine the effects of sunlight exposure of pigs on serum concentration as well as loin and subcutaneous adipose tissue content of 25(OH) and vitamin D3.

## Materials and methods

All experimental protocols were approved by the University of Wyoming (UW) institutional animal care and use committee (IACUC).

White Landrace-Duroc cross pigs (n = 28, 14 male, 14 female; 81±16 d of age, 29±1 kg) were assigned at random to either sunlight exposure (SUN) in the spring and summer (n = 14) or confined to indoors as per standard practice (Control) with limited exposure to outdoor sunlight (n = 14). The study was replicated with a second group of white Yorkshire-Duroc-Landrace cross-bred pigs (n = 24, 10 male, 14 female) aged 80±13 d, 31.6±2.1 kg with sunlight exposure (SUN) in the summer and fall (n = 12) or Control (n = 12). From each group of pigs, a subsample of sun-exposed (n = 5 per group) and control (n = 4 per group) pigs, matched for body condition score, were selected for blood and tissue analysis; these analyses were performed only in a subsample due to cost constraints. Pig breeds selected are commonly used for pork production. Waldo Farms (DeWitt, NE) supplied the spring-summer group of pigs. The summer-fall group of pigs was born to the University of Wyoming Research and Extension Center farm.

Pigs were raised at UW research and extension center in a confined temperature-controlled system with natural airflow and provided standard care including *ad libitum* water and feed. Diets were formulated as a growth ration with a corn and soybean base and vitamin and trace mineral premixes added (nutrient analysis is shown in Table 1). Pigs were housed by group in 3.7 x 3.7 m pens (n = 14 max/pen) with a combination of solid and slotted flooring; the slotted flooring portion of the pens were washed with water twice a day.

**Table 1:** Nutrient content of soybean and corn feed with added vitamin and mineral premixes.

Energy (kcal/kg)	3452
Crude Protein (%)	15.1
Lysine (%)	0.89
Calcium (%)	0.67
Phosphorus^a^ (%)	0.62
Vitamin D_3_^b^ (IU/kg) Vitamin A^b^ (IU/kg) Vitamin E^b^ (IU/kg)	1239 8261 41

Data provided in Kcal/kg of feed with the nutrients shown as the percentage of diet fed as estimated by the National Research Council.

^a^ Monocalcium Phosphate

^b^ Vitamin premix (Trouw Nutrition, Highland, IL) mixed per manufacturer specification.

Spring-summer pigs assigned to sun exposure were exposed to natural sunlight one hour each day during solar noon (12:30–1:30 PM standard time) for two weeks surrounding the spring equinox (March 20–April 3) and summer solstice (June 13–26). Summer-fall pigs were exposed to sunlight for an hour daily during solar noon for two weeks surrounding the summer solstice (June 17–30) and fall equinox (September 18-October 1). Sun pigs traveled down a short alley to an outdoor pen on the southern side of the facility. In both groups of SUN pigs, the first exposure period occurred when the pigs were young growers and the second exposure occurred when the pigs were near slaughter weight. Control pigs were raised conventionally, and kept in confinement, with limited sun exposure, throughout the duration of the experiment. Total UV radiation was measured during the exposure period by using a UV spectrometer (Eppley Laborotories Inc. Newport, RI) and a logging data acquisition system (National Instruments, Austin, TX). UVB was calculated by using a UVA/UVB ratio of 20:1,i.e. 5% of total [24]. Blood samples were obtained via cranial vena cava puncture at the beginning and end of the second exposure period in SUN and Control pigs. Blood samples were allowed to clot for 60 minutes and centrifuged (800 *g* for 20 minutes) by using a Beckman TJ-6 (Brea, CA). Serum was collected and stored at -20°C until analysis.

The selected pigs from spring-summer and summer-fall were slaughtered at the University of Wyoming abattoir on the morning following the last day of sun exposure. At slaughter the pigs were killed by electrical stun followed by exsanguination as per standard slaughter procedures and as approved by the IACUC. Three random samples (≈ 3 g) from a single pork chop (i.e. center loin, tenth rib, and the surroundingadipose) were collected and frozen on dry ice, and stored in eppendorf tubes at -80°C until analysis. Back fat thickness was taken by the average of measurements at the 10^th^ and last rib. Percentage of fat-free lean was calculated by using the equation of Boggs et al [25].

Serum and tissue vitamin D analyses were conducted by Heartland Assays (Ames, IA) who were blinded for treatment group. Serum concentrations of 25(OH)D_3_ were determined by radioimmunoassay (RIA) with tissue content of vitamin D_2_, D_3_ and 25(OH)D determined via liquid chromatography-mass spectrometry (LC/MS/MS).

### Serum 25(OH)D via RIA

The 25(OH)D assay is a two-step procedure [26] using rapid extraction of 25-OH-D and other hydroxylated metabolites with acetonitrile followed by RIA analysis. The RIA utilizes a first antibody co-specific for 25(OH)D_2_ and 25(OH)D_3_. Following incubation at room temperature, antigen-antibody complexes were separated with a second antibody precipitating complex followed by centrifugation. 25(OH)D equivalent values were calculated directly by the γ-radiation counting system via a smooth-spline method [26]. The 25(OH)D assay has a range of 2.5–100 ng/ml with 8% intra- and 10% inter-assay CV.

### 25(OH)D_2_/25(OH)D_3_ and vitamin D_2_/vitamin D_3_ in adipose and meat/tissue

All samples were weighed and saponified with methanolic potassium hydroxide [27] and spiked with deuterated internal standard for each metabolite being measured (vitamin D3(6,19,19-d3) and 25-hydroxyvitamin D3 (6,19,19-d3). Both deuterated and non-deutrated reagents were purchased from Sigma-Aldrich (St. Louis, MO). Following saponification, samples were extracted by using hexane/ethyl acetate (85:15 v:v). The organic layer was dried and reconstituted in hexane/methylene chloride (90:10 v:v) and both 25OH and vitamin D were isolated on a 0.5 gram silica SPE column. HPLC was utilized to further purify samples [27]. After purification, samples were dried in Savant vacuum dryer and then re-constituted into LCMS-grade methanol and LCMS high-purity water both with 0.1% formic acid, and then injected onto an Agilent 1290 HPLC with an Agilent C18 Poroshell Column coupled to an Agilent 6460 Triple-quad mass spec (Quantitative Analysis, Version B.07.00, Agilent Technologies, Santa Clara, CA) with electrospray ionization source (ESI) in positive mode and analyzed by using Masshunter software[27]. Both assays had R^2^ values greater than 0.99. 25(OH)D_2/_D_3_ intra-assay %CV = 5.0% and inter-assay %CV = 8.6% with extraction efficiencies of >68% and assay range of 0.5–312.5ng/g. Vitamin D_2_/D_3_ intra-assay %CV = 9.9% and inter-assay %CV = 11.2% with extraction efficiencies of >64%. Limit of quantification was 0.5ng/g and assay range of 0.5-625ng/g with a limit of detection of 0.1ng/g for all analytes. All values were adjusted for extraction efficiency prior to statistical analysis.

### Statistical procedures

Only serum 25(OH)D_3_ data for the second exposure period when the pigs were close to slaughter are included. Data are presented as mean ± standard error unless otherwise specified. Data were analyzed by using Minitab statistical software version 17 (State College, PA). Two sample t-test with equal or unequal variances were used to compare differences in body weights and body composition and pre-treatment serum 25(OH)D_3_ concentrations between SUN and Control pigs. Repeated Measures ANOVA was used to test for a time (pre versus post sun exposure) by treatment (SUN versus Control) effect on serum 25(OH)D_3_ concentrations. A two-sample t-test with equal or unequal variances was used to test for differences in vitamin D_3_ and 25(OH)D_3_ in loin and subcutaneous adipose from the SUN versus control pigs at slaughter and to test for differences between summer and fall exposure pigs for serum and tissue vitamin D concentrations. Pearson correlation coefficients were used to determine associations between tissues and serum concentrations of vitamin D.

## Results

### Body mass, composition and weight gain

Starting weights, slaughter weights, and average daily gain (ADG) were not different between control and SUN pigs for spring-summer and summer-fall groups (Table 2, S1 Table). At slaughter, body composition measured as back fat thickness at the first and last ribs and percentage fat-free lean tissue were similar between control and SUN pigs (Table 2, S1 Table).

**Table 2:** Body weight and body composition in the subgroup of sun-exposed and control pigs selected for tissue analysis.

	Spring/Summer			Summer/Fall		
	Control	SUN	P	Control	SUN	P
Initial Weight (kg)	33.8 ± 2.2	28.9 ± 2.2	0.17	34.4 ± 2.8	28.6 ± 2.7	0.18
ADG (kg/d)	0.90 ± 0.04	0.90 ± 0.05	0.59	0.70 ± 0.05	0.60 ± 0.06	0.44
Slaughter Weight (kg)	118.7 ± 6.0	117.6 ± 3.4	0.81	106.4 ±7.4	93.3 ± 5.6	0.21
Fat-Free Lean (%)	51.3 ± 0.7	52.7 ± 1.1	0.29	58.9 ± 2.5	58.9 ± 2.6	1

Starting weights were taken on same day as first blood sample collected before first sun exposure. Slaughter weights were measured immediately before slaughter. Average daily gain in kg/d was also calculated, covering the entire study period. Weights are presented as means ± the standard error. Fat-free lean percentage was calculated as noted in Boggs et al.

### UV exposure

**Fig 1:**
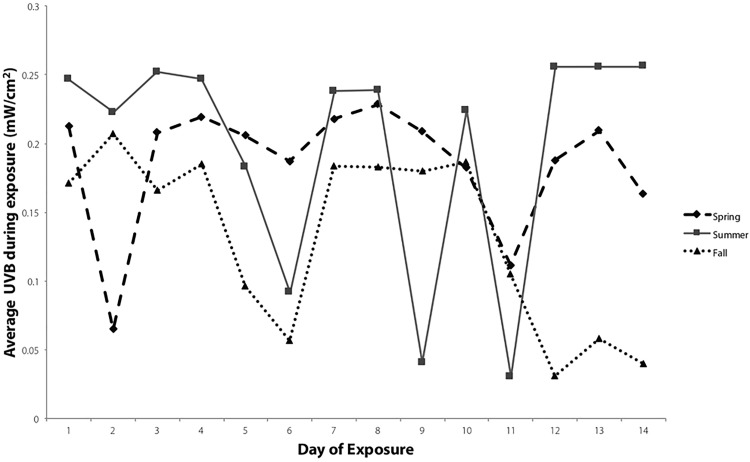
Summer and fall average UVB exposure. Average UVB radiation during noontime sun exposure for 14 days in the summer and fall at close to the summer solstice and fall equinox, respectively. Data were recorded every second for the one hour exposure period around solar noon (± 30 minutes). Each point represents the mean of the 3600 points. Error, expressed as 95% confidence intervals about the mean are not visible (range = 6.2 X 10^−5^ to 0.033731). Days with low UVB radiation were cloudy during the sun exposure period.

Average UVB radiation at solar noon (±30 minutes) for the 14 days of exposure around the summer solstice and 14 days around the fall equinox is shown in Fig 1. Average UVB during the 14-day exposure was higher in the summer compared to the fall (P = 0.009).

### Serum 25(OH)D_3_

**Fig 2:**
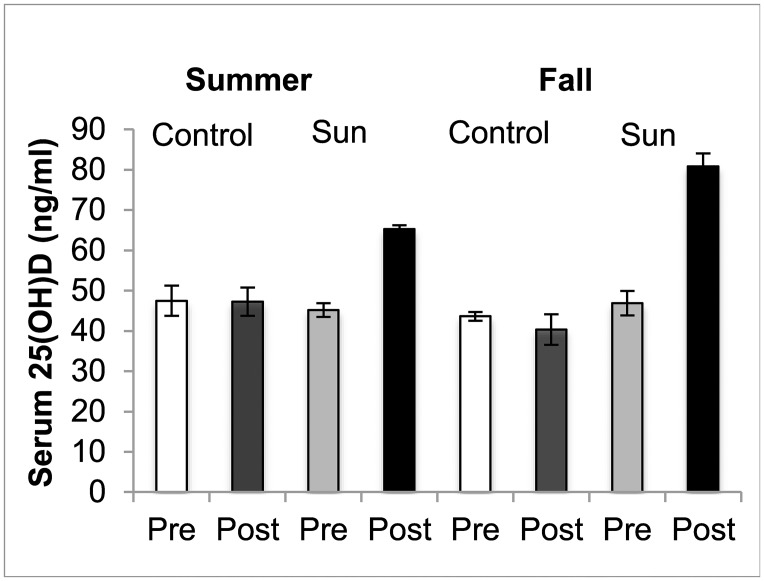
Effects of sun exposure on vitamin D status. Serum 25(OH)D_3_ concentrations before (Pre) and after (Post) sun exposure for one hour daily at solar noon for 2-weeks (SUN) or standard confinement (Control) in the summer and fall. Summer exposure occurred in the spring-summer group and fall exposure occurred in the summer-fall group in finisher pigs 2-weeks prior to slaughter.

There were no differences (P≥0.325) in serum concentrations of 25(OH)D_3_ between SUN (46.1±1.7 ng/ml) and Control pigs (39.9±5.6 ng/ml) prior to sun exposure. Serum 25(OH)D_3_, however, was increased (P<0.001) following sun exposure in SUN but not Control pigs (P<0.001, time*treatment interaction [S1 Fig]) (Fig 2), and was higher post-exposure in the SUN pigs exposed in the fall compared those exposed in the summer (Fig 2).

### Tissue vitamin D

#### Loin tissue

**Fig 3:**
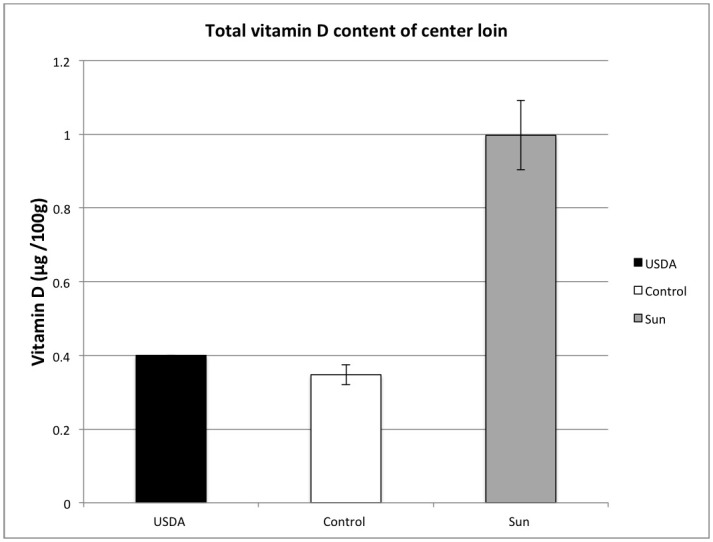
Effects of sun exposure on vitamin D concentrations in loin tissue. Total vitamin D content includes both vitamin D_3_ and 25(OH)D_3_. The black bar represents the value presented in the USDA national nutrient database for the vitamin D content of “raw pork, center loin.” Center loin samples were collected on the day of slaughter. Error bars represent the standard error. (1 ug vitamin D_3_ = 40 IU).

Following sun exposure, vitamin D_3_ content was higher (P<0.001, [S2 Fig]) in the loin of SUN pigs (0.716± 0.097 μg/100g; 28.6± 3.9 IU/100g) compared to control pigs (0.218± 0.024 μg/100g; 8.7± 1.0 IU/100g) as was loin content of 25(OH)D_3_ (SUN = 0.281± 0.014, control = 0.130± 0.016 μg /100g). Total vitamin D content (D3 plus 25(OH)D_3_) of loin was also higher (P = 0.001) in SUN pigs (0.997±0.094 μg/100g) compared to control pigs (0.348±0.027 μg/100g) (Fig 3).

#### Subcutaneous adipose

Adipose content of 25(OH)D_3_ content was greater in SUN pigs (0.814±0.1 μg /100g) versus that in control pigs (0.406±0.044 μg /100g; P = 0.003 [S2 Fig]). Vitamin D_3_ content was not different between groups (SUN = 4.6±0.6 μg/100g; 184.2±25.7 IU/100g; Control = 4.1±0.0.5 μg/100g; 164.3±19.6 IU/100g, P = 0.56). Total vitamin D content in subcutaneous adipose, as well, did not differ (P = 0.35) between SUN (5.42 μg/100g) and control (4.513 μg/100g) pigs.

Vitamin D_2_ was not detectable in any tissue samples.

### Association between serum 25(OH)D_3_ and tissue concentrations

Serum 25(OH)D_3_ concentrations were positively associated with loin vitamin D_3_ (r = 0.63, P = 0.005) loin 25(OH)D_3_ (r = 0.83, P<0.001) and subcutaneous fat 25(OH)D_3_ (r = 0.73, P = 0.001) content, but not with subcutaneous adipose vitamin D_3_ content (r = 0.19, P>0.05).

## Discussion

We have demonstrated sun exposure prior to slaughter in pigs significantly increases serum concentrations of vitamin D and vitamin D content in the lean tissue of the loin. To our knowledge, this is the first study demonstrating the effects of natural sunlight in pigs on the vitamin D content of pork products.

The main finding of this experiment is of significant importance when considering dietary intake of vitamin D. The current recommended dietary allowance (RDA) for vitamin D is 600 IU/d for children and adults <70 years old [28]. Despite a recent increase in the RDA of vitamin D from 400 IU to 600 IU, many researchers believe the current RDA is still not sufficient for optimal health [29,30]. For example, the Endocrine Society recommends 1500–2000 IU/d of vitamin D for most individuals with higher intakes recommended for individuals at risk for vitamin D deficiency [30]. While the RDA of 600 IU/d supports circulating 25(OH)D_3_ concentrations of 20 ng/ml (sufficient to prevent clinical rickets) such circulating concentrations may not provide further health benefits including reduced risk of certain cancers [31,32,33] and prevention of stress fractures [34]. Heaney suggests the optimal concentration of 25(OH)D_3_ should be at least 30 ng/ml and further stresses that maintaining circulating concentrations of 25(OH)D_3_ in the range of 30–50 ng/ml may produce added health benefits [35]. Furthermore, there is evidence that a concentration of 48 ng/ml 25(OH)D_3_ may be optimal for many physiological functions such as fully suppressed parathyroid hormone, efficient intestinal calcium absorption, and optimized vitamin D concentrations in breastmilk [8]. These higher concentrations of 25(OH)D_3_ are believed to approach those of our hominid ancestors based on research in African tribes [36]. Considering this evidence, increased vitamin D in the diet may be beneficial for achieving optimal 25(OH)D_3_ status, or at a minimum, meeting the RDA for vitamin D. Many people in the US [10] and worldwide [9] fail to maintain vitamin D concentrations above 20 ng/ml.

Strategies to fortify or naturally add vitamin D to the food supply are currently being explored. Green and colleagues [37] have suggested a greater range of food vehicles for fortification, other than dairy, margarine, and cereals, may be necessary to improve the vitamin D status of populations. This increase in dietary vitamin D may be achieved by enhancing the natural vitamin D content of foods, termed bio-addition. Current strategies of bio-addition include exposing mushrooms to UV light after harvest [38], and supplementing laying hens with vitamin D to increase the vitamin D content of egg yolks [39]. Altering the vitamin D content of feed has also been demonstrated to affect the vitamin D_3_ and 25(OH)D_3_ content in meat cuts from pigs[40]. Altering modern swine management practices to allow pigs exposure to sunlight may also be an effective means to naturally increase the vitamin D content of pork products.

A recent study found that exposing hens to artificial sunlight via 23-W UVB lamps for 3 hr daily for four weeks increased the vitamin D content of egg yolks by 337% and the content of fibularis longus muscle to detectable concentrations of 0.16 to 0.96 μg/100g [23]. This UVB exposure protocol was three to four times more effective as supplementation with 3000 IU of vitamin D/kg of feed in increasing D_3_ concentrations of egg yolks and meat. Another study in a small sample of Gottingen minipigs reported that UVB exposure was more effective than dietary administration of vitamin D_3_ at increasing vitamin D tissue content [21]. Our data support exposure to natural sunlight for an hour per day is effective at increasing vitamin D content of pork products.

In a previous study of Clausen and colleagues [41], a thorough analysis of vitamin D content of pork cuts in pigs conventionally raised in absence of direct sunlight exposure yielded a vitamin D_3_ and 25(OH)D, content of 0.4 and 0.5 ng/g in the loin tissue, and 5 and 3.1 ng/g in the adipose, respectively. In contrast, sun-exposed pigs in our experiment had an average D_3_ content of 7.16 ng/g in the loin and 46.05 ng/g in subcutaneous adipose. This increase is a near 18-fold of D_3_ in loin tissue and a 9-fold increase in subcutaneous adipose tissue. In the present experiment 25(OH)D content averaged 2.81 ng/g (11.2 IU/100g) in the loin and 8.14 ng/g (32.6 IU/100g) adipose of sun-exposed pigs. Interestingly, our control pigs also had higher vitamin D content than pigs in the Clausen et al. analysis, which is likely attributable to the dietary supplementation of our pigs. The USDA nutrient database reports the vitamin D_3_ content of a raw, lean center loin to be 14 IU/100g. Our sun-exposed pigs had an average total vitamin D content of 39.9 IU/100g. The USDA nutrient database currently does not include the 25(OH)D_3_ content of meat products. Considering that 25(OH)D_3_ is present in natural animal-based products, and has five times the biological activity of vitamin D_3_, 25(OH)D_3_ should be measured and accounted for in future vitamin D analysis.

Interestingly, in the current study, the vitamin D_3_ content of subcutaneous adipose was not further improved by sun exposure and was similar between the sun-exposed and control pigs, despite a 100% percent increase in 25(OH)D_3_. The reason for this is unclear. It is possible that a ceiling effect for D_3_ storage in adipocytes was reached by the content of D_3_ in feed which produced near optimal serum concentrations even before sun exposure (i.e., >40 ng/ml), and could not be further increased with sun exposure. Another possibility is that 25(OH)D_3_ concentrations increased in subcutaneous adipose as a direct result of higher serum 25(OH)D_3_, which may enhance adipose hydroxylation of 25(OH)D_3_. Further research is needed to address these possibilities.

This study has shown that daily sun exposure for two weeks in pigs prior to slaughter increases the vitamin D content of the loin. Lean pork, recently shown to be an effective replacement for chicken and fish in the DASH (Dietary Approaches to Stop Hypertension) diet, can be an additional source of dietary vitamin D especially for individuals with high blood pressure [42]. This research has provided insight into the regulation of vitamin D content in pork products, and the possible benefits of raising pigs with controlled exposure to sunlight. Future work should confirm these findings in other breeds of pigs and determine if shorter, less frequent sunlight exposures would be equally as effective at enhancing vitamin D content of loin and other pork products.

## Supporting information

S1 Table Body weight and body composition in the subgroup of sun-exposed and control pigs selected for tissue analysis. Starting weights were taken on same day as first blood sample collected before first sun exposure. Slaughter weights were measured immediately before slaughter. Average daily gain in kg/d was also calculated, covering the entire study period. Weights are presented as means ± the standard error. Fat-free lean percentage was calculated as noted in Boggs et al.
(XLSX)

S1 Fig Effects of sun exposure on vitamin D status. Serum 25(OH)D_3_ concentrations before (Pre) and after (Post) sun exposure for one hour daily at solar noon for 2-weeks (SUN) or standard confinement (Control) in the summer and fall. Summer exposure occurred in the spring-summer group and fall exposure occurred in the summer-fall group in finisher pigs 2-weeks prior to slaughter.
(XLSX)

S2 Fig Effects of sun exposure on vitamin D concentrations in loin tissue. Total vitamin D content includes both vitamin D_3_ and 25(OH)D_3_. The black bar represents the value presented in the USDA national nutrient database for the vitamin D content of “raw pork, center loin.” Center loin samples were collected on the day of slaughter. Error bars represent the standard error. (1 ug vitamin D_3_ = 40 IU).
(XLSX)
